# Genetic parameters of five new European Standard Set STR loci (D10S1248, D22S1045, D2S441, D1S1656, D12S391) in the population of eastern Croatia

**DOI:** 10.3325/cmj.2012.53.409

**Published:** 2012-10

**Authors:** Goran Ćurić, Vedran Gašić, Vera Plužarić, Danijela Smiljčić

**Affiliations:** 1 DNA Laboratory, School of Medicine, J. J. Strossmayer University, Osijek, Croatia; 2 Department of Pathology and Legal Medicine, Clinical Hospital Osijek, Osijek, Croatia; 3 School of Medicine, J. J. Strossmayer University, Osijek, Croatia; 4 Clinic of Dermatology and Venerology, Clinical Hospital Osijek, Osijek, Croatia; 5 Worldwide Clinical Trials Ltd., Zagreb, Croatia

## Abstract

**Aim:**

To establish allele frequencies and genetic parameters in eastern Croatia population and to compare them with those in other populations. The second aim was to compare the genetic profiles obtained with different forensic kits amplifying the same genetic markers.

**Methods:**

Blood samples of 217 unrelated individuals from eastern Croatia were genotyped using AmpFlSTR NGM kit. Allele distribution and other genetic parameters were determined for 15 short tandem repeat (STR) loci, including the 5 loci recently added to the European Standard Set (ESS) of STR loci (D10S1248, D22S1045, D2S441, D1S1656, and D12S391). Ninety-six samples underwent duplicate analysis using AmpFlSTR Identifiler kit.

**Results:**

Power of discrimination was highest for the two new ESS loci, D1S1656 (0.97254) and D12S391 (0.97339). Comparison of allele frequencies for 5 new ESS loci in our sample with previously published population data showed a significant difference from Maghreb population on D2S441 and from American Caucasian population on D1S1656. Comparison of allele frequencies for standard 10 STR loci with all the neighboring populations’ data showed a significant difference only from Albanian population (on D2S1338, D18S51, and TH01). Discordant genotypes were observed in 5 (5.2%) samples at a single locus when amplified with both AmpFlSTR NGM and AmpFlSTR Identifiler kit.

**Conclusion:**

New ESS STR loci are highly polymorphic and short, and therefore very useful for the analysis of challenging forensic samples. DNA samples purposed for establishing databases should be routinely amplified in duplicate.

To facilitate DNA profiles comparison between databases of different European countries, The European Network of Forensic Science Institutes (ENFSI) and European DNA Profiling Group (EDNAP) have recently added five new loci (D10S1248, D22S1045, D2S441, D1S1656, and D12S391) to the European Standard Set of short tandem repeat (STR) loci (1,2). These new loci were included into the AmpFlSTR NGM PCR amplification kit (NGM kit; Life Technologies, Foster City, CA, USA).

The Laboratory for DNA Analysis in Osijek was established to participate in the identification of missing persons after the war in Croatia (1991-1995). In collaboration with the laboratories in Zagreb and Split, a database of genotypes of missing persons’ relatives was created including approximately 5000 persons. The greatest part of the included genetic information is based on the 15 loci incorporated in AmpFlSTR Identifiler PCR amplification kit (Identifiler kit; Life Technologies, Foster City, CA, USA). Skeletal remains are identified by comparing the genotype of each piece of skeletal remains with the genotypes in the missing persons’ relatives database. Such non-targeted matching in a database containing several thousands genotypes considerably decreases the reliability of the established match. Still, the majority of identified skeletal remains were matched in such a way, as genotypes of the missing persons from the father-mother-child trio. Even within so large a database, hundreds of genotypes of skeletal remains still do not have a match, due to a lack of adequate relatives. Matching a profile created from a piece of skeletal remains across the whole database returns many adventitious matches, partly because some genotyped loci have low discrimination power (2). An especially large number of adventitious matches is present if the genetic profile from skeletal remains is partial.

In a targeted approach to DNA typing, loci on the Y-chromosome and mtDNA can be amplified, but at a database level more useful are the loci on somatic chromosomes. Evidential value of a genetic match based on STR typing relies on high polymorphism and a large number of STR loci. In order to obtain as much as possible genetic information, we used the NGM kit. Our aim was to increase the number of genetic markers in order to achieve higher evidential value of STR typing and to amplify short STR loci, often better preserved in degraded samples. Especially valuable are three new “mini” STR loci (D10S1248, D22S1045, and D2S441), engineered to produce short amplicons (up to 150 bp) that are more successfully obtained from the most degraded samples. The remaining two new loci (D1S1656 and D12S391) are also relatively short and highly polymorphic (3-7). Besides obtaining information on the 5 new loci, the NGM kit includes improved chemistry that maximizes performance on challenging samples.

In the new European Standard Set (ESS) of STR loci, allele distribution and genetic parameters still have to be determined. A population study on the new loci has been performed for several countries (including Belgium, Germany, Hungary, Maghreb countries, Poland, and USA) (7-12). However, there has been no such study either for Croatian or its neighboring populations. Therefore, we carried out a population study on a sample from eastern Croatia, which might be the most appropriate regional sample, because this part of the country sustained the greatest human losses during the war. Since the greatest part of our relatives’ database is based on the Identifiler kit, which shares 10 loci with NGM kit (D3S1358, vWA, D16S539, D2S1338, D8S1179, D21S11, D18S51, D19S433, TH01, and FGA), we compared the genetic profiles obtained with both of these kits amplifying the same genetic markers. We also compared the obtained genetic parameters for 15 STR loci with the available population data from the neighboring countries.

## Materials and methods

### Sampling and extraction

This study was conducted on a population sample of 217 unrelated persons from eastern Croatia (population of approximately 500 000 inhabitants). A study based on 100-150 participants is generally accepted as appropriate for determining population data (13). The tested individuals were voluntary donors and gave informed consent. Blood samples were anonymized prior to analyses. The study was conducted in DNA Laboratory of School of Medicine of J. J. Strossmayer University, Osijek, Croatia, during the summer of 2011. The research project was approved by the Medical Ethics Committee of the J. J. Strossmayer University in Osijek. Genomic DNA was extracted from Whatman FTA Bloodstain Card (Whatman, Florham Park, NJ, USA) using Instagene chemistry (Bio-Rad Laboratories, Hercules, CA, USA), according to the manufacturer’s protocol. The quantity of DNA was determined using Quantifiler Human DNA Quantification Kit in ABI Prism 7000 Sequence Detection System Instrument (both from Life Technologies), according to the manufacturer’s recommendations.

### Genotyping

DNA was amplified using AmpFlSTR NGM PCR amplification kit (Life Technologies) in all samples (amplified loci: D3S1358, vWA, D16S539, D2S1338, D8S1179, D21S11, D18S51, D19S433, TH01, FGA, D10S1248, D22S1045, D2S441, D1S1656, D12S391, and amelogenin) and with AmpFlSTR Identifiler PCR amplification kit (Life Technologies) in samples from 96 participants. A multiplex DNA amplification was carried out in a Perkin-Elmer thermo cycler (Life Technologies), according to the manufacturer’s recommendations. Electrophoresis was performed on the ABI PRISM® 310 Genetic Analyzer (Life Technologies), data were analyzed using GeneMapperID version 3.2 (Life Technologies), and STR allele designations were made based on the comparison with the appropriate allelic ladder. All samples with peak imbalance and questionable allele calls were reanalyzed. Control DNA 007 included in the NGM kit was used as positive control. GEDNAP quality control proficiency testing was conducted in DNA Laboratory of School of Medicine of J. J. Strossmayer University, Osijek, Croatia.

### Statistical analysis

Allele frequencies for 15 loci and statistical parameters of forensic interest: observed and expected heterozygosity (H_obs_, H_exp_), standard error, and Hardy-Weinberg equilibrium (HWE) were calculated with the ARLEQUIN software, version 3.5.1.3 (14). We used the same software to compare the allele frequencies with the previously published population data for the new ESS loci (Belgium, Germany, Hungary, Maghreb, Poland, and USA) (7-12) and for the remaining 10 STR loci (Croatia, Albania, Bosnia, Hungary, Macedonia, Serbia and Montenegro, and Slovenia) (15-21). Power of discrimination (PD), power of exclusion (PE), polymorphism information content (PIC), and typical paternity index (TPI) were calculated using PowerStats, version 12 (Promega, Fitchburg, WI, USA).

## Results

The two new ESS loci, D1S1656 and D12S391, had the highest power of discrimination (0.97254 and 0.97339, respectively), as well as PIC (0.88068 and 0.88045, respectively) (Table 1). Deviation from Hardy-Weinberg equilibrium in case of vWA (0.04060), D16S539 (0.01214), and D21S11 STR locus (0.03974) was observed, but rejected after Boniferroni correction (Table 1). For five new ESS loci, our sample showed a significant difference in allele frequencies only from Maghreb population on D2S441 (*P* < 0.001) and from American Caucasian population on D1S1656 (*P* = 0.003) (Table 2). For the remaining 10 loci, allele frequencies included in NGM kit were compared with the previously published population data for Croatia, Albania, Bosnia, Hungary, Macedonia, Serbia and Montenegro, and Slovenia and significant differences were found for Albanian population on the locus D2S1338 (*P* = 0.022), D18S51 (*P* = 0.029), and TH01 (*P* = 0.006) (Table 3).

**Table 1:** Allele frequencies and statistical parameters of 15 short tandem repeat (STR) loci amplified with AmpFlSTR NGM PCR amplification kit in a population sample from eastern Croatia (N = 217)*

Loci	D10S1248	vWa	D16S539	D2S1338	D8S1179	D21S11	D18S51	D22S1045	D19S433	TH01	FGA	D2S441	D3S1358	D1S1656	D12S391
**6**	-	-	-	-	-	-	-	-	-	0.21114	-	-	-	-	-
**7**	-	-	-	-	-	-	-	-	0.00230	0.16009	-	-	-	-	-
**8**	-	-	0.01617	-	0.01613	-	-	-	-	0.09281	-	0.00230	-	-	-
**8,3**	-	-	-	-	-	-	-	-	-	0.00464	-	-	-	-	-
**9**	-	-	0.11547	-	0.00922	-	-	-	-	0.18329	-	-	-	-	-
**9,3**	-	-	-	-	-	-	-	-	-	0.34107	-	-	-	-	-
**10**	-	0.00460	0.04619	-	0.03687	-	0.00924	0.00461	0.00230	0.00696	-	0.21198	-	0.00230	-
**11**	0.00461	0.00691	0.30485	-	0.07834	-	0.00924	0.15207	0.01382	-	-	0.30184	-	0.08986	-
**11,3**	-	-	-	-	-	-	-	-	-	-	-	0.06221	-	-	-
**12**	0.04147	-	0.31178	-	0.18894	-	0.13164	0.01843	0.08295	-	-	0.03456	0.00230	0.14977	-
**13**	0.23041	0.00460	0.17552	-	0.32488	-	0.13857	-	0.23272	-	-	0.02995	0.00691	0.04608	-
**13,2**	-	-	-	-	-	-	-	-	0.01843	-	-	-	-	-	-
**14**	0.30415	0.12211	0.03002	-	0.18894	-	0.15935	0.06221	0.33180	-	-	0.31106	0.12673	0.08986	-
**14,2**	-	-	-	-	-	-	-	-	0.02535	-	-	-	-	-	-
**15**	0.2212	0.12211	-	-	0.11290	-	0.16628	0.34793	0.16590	-	-	0.04608	0.21659	0.14055	0.03226
**15,2**	-	-	-	-	-	-	-	-	0.04608	-	-	-	-	-	-
**15,3**	-	-	-	-	-	-	-	-	-	-	-	-	-	0.04147	-
**16**	0.15207	0.19124	-	0.06019	0.04378	-	0.12240	0.32949	0.04839	-	0.00230	-	0.26267	0.13134	0.02304
**16,2**	-	-	-	-	-	-	-	-	0.02074	-	-	-	-	-	-
**16,3**	-	-	-	-	-	-	-	-	-	-	-	-	-	0.03687	-
**17**	0.03456	0.24193	-	0.22454	-	-	0.10855	0.08295	0.00230	-	0.00691	-	0.19355	0.05991	0.10829
**17,2**	-	-	-	-	-	-	-	-	0.00230	-	-	-	-	-	-
**17,3**	-	-	-	-	-	-	-	-	-	-	-	-	-	0.15207	0.00691
**18**	0.01152	0.20967	-	0.08565	-	-	0.07159	0.00230	-	-	0.01382	-	0.18203	0.01382	0.16359
**18,2**	-	-	-	-	-	-	-	-	0.00461	-	-	-	-	-	-
**18,3**	-	-	-	-	-	-	-	-	-	-	-	-	-	0.03917	0.02074
**19**	-	0.08064	-	0.12963	-	-	0.05081	-	-	-	0.08986	-	0.00922	-	0.11521
**19,3**	-	-	-	-	-	-	-	-	-	-	-	-	-	0.00230	0.02074
**20**	-	0.01382	-	0.15972	-	-	0.01848	-	-	-	0.12442	-	-	-	0.12903
**20,2**	-	-	-	-	-	-	-	-	-	-	0.00230	-	-	-	-
**20,3**	-	-	-	-	-	-	-	-	-	-	-	-	-	0.00461	0.00230
**21**	-	0.00230	-	0.02083	-	-	0.00693	-	-	-	0.19124	-	-	-	0.11751
**22**	-	-	-	0.01852	-	-	0.00693	-	-	-	0.20968	-	-	-	0.13594
**22,2**	-	-	-	-	-	-	-	-	-	-	0.00691	-	-	-	-
**23**	-	-	-	0.07870	-	-	-	-	-	-	0.14055	-	-	-	0.07143
**23,2**	-	-	-	-	-	-	-	-	-	-	0.01613	-	-	-	-
**24**	-	-	-	0.08796	-	-	-	-	-	-	0.10369	-	-	-	0.02765
**24,2**	-	-	-	-	-	-	-	-	-	-	0.00461	-	-	-	-
**25**	-	-	-	0.11574	-	-	-	-	-	-	0.06912	-	-	-	0.01613
**26**	-	-	-	0.01852	-	0.00461	-	-	-	-	0.01613	-	-	-	-
**27**	-	-	-	-	-	0.03226	-	-	-	-	0.00230	-	-	-	0.00230
**28**	-	-	-	-	-	0.17512	-	-	-	-	-	-	-	-	-
**29**	-	-	-	-	-	0.22811	-	-	-	-	-	-	-	-	-
**29,2**	-	-	-	-	-	0.00230	-	-	-	-	-	-	-	-	-
**30**	-	-	-	-	-	0.20507	-	-	-	-	-	-	-	-	-
**30,2**	-	-	-	-	-	0.04608	-	-	-	-	-	-	-	-	-
**31**	-	-	-	-	-	0.05760	-	-	-	-	-	-	-	-	-
**31,2**	-	-	-	-	-	0.09447	-	-	-	-	-	-	-	-	-
**32**	-	-	-	-	-	0.01152	-	-	-	-	-	-	-	-	-
**32,2**	-	-	-	-	-	0.09908	-	-	-	-	-	-	-	-	-
**33,2**	-	-	-	-	-	0.03687	-	-	-	-	-	-	-	-	-
**34,2**	-	-	-	-	-	0.00691	-	-	-	-	-	-	-	-	-
**H_obs_**	0.82949	0.80645	0.69907	0.84722	0.83410	0.89862	0.84722	0.82028	0.81567	0.78140	0.85253	0.79724	0.81567	0.90783	0.86175
**H_exp_**	0.78108	0.82622	0.76450	0.86988	0.80240	0.85042	0.87741	0.73784	0.79708	0.77277	0.86176	0.76086	0.79914	0.89280	0.89262
**HWe**	0.29105	0.04060	0.01214	0.81202	0.90304	0.03974	0.83721	0.13598	0.91345	0.61966	0.21165	0.40154	0.85221	0.12752	0.10163
**SE**	0.00037	0.00016	0.00011	0.00025	0.00029	0.00016	0.00030	0.00031	0.00021	0.00038	0.00027	0.00039	0.00033	0.00025	0.00023
**PD**	0.90641	0.94035	0.90844	0.96755	0.93003	0.95033	0.96969	0.86258	0.92947	0.91037	0.96090	0.89601	0.92412	0.97254	0.97339
**PE**	0.65487	0.61107	0.42683	0.68940	0.66378	0.79260	0.68940	0.63720	0.62844	0.56498	0.69987	0.59392	0.62844	0.81145	0.71815
**PIC**	0.74487	0.80063	0.72557	0.85422	0.77466	0.83110	0.86232	0.69355	0.76918	0.73600	0.84422	0.72078	0.76593	0.88068	0.88045
**TPI**	2.93	2.58	1.66	3.27	3.01	4.93	3.27	2.78	2.71	2.29	3.39	2.47	2.71	5.43	3.62

*Abbreviations: H_obs_ – observed heterozygosity; H_exp_ – expected heterozygosity; HWE – probability value of the Hardy-Weinberg equilibrium exact test; SE – standard error; PD – power of discrimination; PE – power of exclusion; PIC – polymorphism information content; TPI – typical paternity index. Five new European Standard Set STR loci are underlined.

**Table 2:** Comparison of allele frequencies on five new European Standard Set loci between Croatian population and previously published population data*

	Short tandem repeat loci (exact test ± standard error)				
Population	D10S1248	D22S1045	D2S441	D1S1656	D12S391
**Maghreb (****7****)**	0.25473 ± 0.0088	0.12696 ± 0.0135	**0.00028 ± 0.0002**	0.12532 ± 0.0083	0.60092 ± 0.0101
**Belgian (****8****)**	0.40503 ± 0.0169	0.55471 ± 0.0148	0.69436 ± 0.0153	0.84137 ± 0.0087	0.89163 ± 0.0081
**Germany (****9****)**	0.51554 ± 0.0106	0.55162 ± 0.0167	0.72850 ± 0.0129	0.38818 ± 0.0255	0.74654 ± 0.0120
**Hungary (****10****)**	0.85019 ± 0.0083	0.61013 ± 0.0202	0.97941 ± 0.0032	0.61322 ± 0.0160	0.97893 ± 0.0034
**Poland (****11****)**	0.72678 ± 0.0096	0.92488 ± 0.0059	0.93853 ± 0.0050	0.65464 ± 0.0121	0.94195 ± 0.0060
**America (****12****)**	0.72580 ± 0.0098	0.45151 ± 0.0115	0.96235 ± 0.0019	**0.00290 ± 0.0008**	0.77636 ± 0.0169

*P value of the exact test of population differentiation. Significant differences (*P* < 0.05) are bold.

**Table 3:** Comparison of allele frequencies on 10 standard European Standard Set loci between Croatian population and previously published population data of neighboring countries*

Short tandem repeat loci (exact test ± standard error)										
Population	vWa	D16S539	D2S1338	D8S1179	D21S11	D18S51	D19S433	TH01	FGA	D3S1358
**Croatia (****15****)**	0.98791 ± 0.0026	0.98261 ± 0.0016	0.97780 ± 0.0032	0.32128 ± 0.0138	0.86963 ± 0.0083	0.32533 ± 0.0177	0.88619 ± 0.0070	0.48746 ± 0.0145	0.65950 ± 0.0244	0.68583 ± 0.0134
**Albania (****16****)**	0.93214 ± 0.0054	0.86350 ± 0.0073	**0.02205 ± 0.0033**	0.14248 ± 0.0121	0.24525 ± 0.0100	**0.02905 ± 0.0035**	0.12218 ± 0.0062	**0.00600 ± 0.0016**	0.54659 ± 0.0145	0.65175 ± 0.0071
**Bosnia (****17****)**	0.73686 ± 0.0129	0.19586 ± 0.0039	-	0.81754 ± 0.0081	0.91468 ± 0.0036	0.99117 ± 0.0009	-	0.34811 ± 0.0090	0.34608 ± 0.0184	0.92274 ± 0.0066
**Hungary (****18****)**	0.13889 ± 0.0072	0.82106 ± 0.0091	0.86182 ± 0.0086	0.57061 ± 0.0180	0.99931 ± 0.0006	0.97975 ± 0.0017	0.80007 ± 0.0048	0.63630 ± 0.0156	0.59924 ± 0.0401	0.68309 ± 0.0188
**Macedonia (****19****)**	0.93284 ± 0.0048	0.87959 ± 0.0077	0.69522 ± 0.0128	0.16556 ± 0.0076	0.89745 ± 0.0067	0.97623 ± 0.0014	0.94138 ± 0.0042	0.10913 ± 0.0097	0.84246 ± 0.0058	0.57256 ± 0.0091
**Serbia and Montenegro (****20****)**	0.99771 ± 0.0006	0.89693 ± 0.0038	0.82378 ± 0.0074	0.89006 ± 0.0049	0.95605 ± 0.0028	0.90717 ± 0.0078	0.89030 ± 0.0069	0.38505 ± 0.0079	0.80217 ± 0.0093	0.64783 ± 0.0130
**Slovenia (****21****)**	0.78836 ± 0.0108	0.97187 ± 0.0022	0.98439 ± 0.0028	0.20882 ± 0.0144	0.99405 ± 0.0009	0.99575 ± 0.0012	0.96367 ± 0.0039	0.25449 ± 0.0233	0.99584 ± 0.0009	0.89045 ± 0.0055

*P value of the exact test of population differentiation. Significant differences (*P* < 0.05) are bold.

## Discussion

We found that new ESS STR loci in Croatian population were highly polymorphic, which is in line with the previously published data about these loci (7-12).

When allele frequencies of 10 standard ESS loci for Croatian population were compared with Albania, Bosnia, Hungary, Macedonia, Serbia and Montenegro, and Slovenia (15-21), significant differences were found only for Albanian population. Such lack of significant differences is in line with the history of population migrations on the Balkans in the last centuries (22,23).

Since there were no significant differences in the allele frequencies of 10 STR loci between our and the neighboring populations, it might be concluded that there are no significant differences for the new STR loci too. This assumption should be tested in future research, but due to lack of population data for the new ESS loci, our population data might serve as a rough approximation of the population data in these countries.

Analysis of more STR loci increases the discrimination power. Independent assortment of STR markers on the same chromosome cannot be assumed and a complex kinship analysis requires an increased number of loci across different chromosomes. Therefore, in case of syntenic loci it is recommended to use a more informative locus in a specific case of probability calculation (12). The NGM kit includes two pairs of syntenic loci, vWA and D12S391 on the chromosome 12, and D2S1338 and D2S441 on the chromosome 2. When syntenic loci were excluded from the kinship analysis, the probability calculation was based on 13 out of 15 loci. The probability calculation based on 13 loci in non-targeted matching approach might be insufficient for reliable identification, ie, in cases of reverse uniparental testing, especially if mutation event is also included in the calculation. When samples were amplified with both NGM and Identifiler kit, 16 non-syntenic out of 20 loci were used for probability calculation. DNA profiling based on 20 loci has very high discriminatory power (24) and should enable reliable identification even in the complex relationship testing.

Our relatives’ database is based on 15 loci included in the Identifiler kit, 10 of which are also included in the NGM kit. In order to identify discordant genotypes, we performed a duplicate analysis for 96 samples. We observed 5 (5.2%) discordant genotypes and in each case allelic dropout occurred when samples were amplified with NGM kit. Non-amplification of the second allele in a heterozygous genotype was observed for the D18S51 (once), D2S1338 (twice), and D21S11 (twice), and it was probably a result of random nature of PCR. Therefore, DNA samples purposed for establishing genotype databases should be routinely amplified in duplicate.

In a non-targeted approach to matching, when the genotype of each piece of skeletal remains is compared to thousands of genotypes in the database, false inclusion is common. In our experience, in about 5% of the cases more than 5 potential parents or children match the genotype of a specific piece of skeletal remains on 15 loci. Experience taught us not to “believe” the DNA without correspondence with other forensic information, like place of disappearance or matching personal traits or belongings. Non-targeted matching through large databases should never rely solely on genetic information, and anthropologic or other forensic data should be taken into account.
